# Relationship between night shift and sleep problems, risk of metabolic abnormalities of nurses: a 2 years follow-up retrospective analysis in the National Nurse Health Study (NNHS)

**DOI:** 10.1007/s00420-023-02014-2

**Published:** 2023-10-24

**Authors:** Heli Zhang, Jingpin Wang, Siwei Zhang, Sumei Tong, Jinping Hu, Ying Che, Lin Zhuo, Peng Wang, Rongmei Geng, Yujie Zhou, Panfeng Wang, Siyan Zhan, Baohua Li

**Affiliations:** 1 Department of Rehabilitation Medicine, Peking University Third Hospital, 49 North Garden Rd., Haidian District, Beijing, 100191 People’s Republic of China; 2 Nursing Department, Peking University Third Hospital, 49 North Garden Rd., Haidian District, Beijing, 100191 People’s Republic of China; 3 Department of Cardiology, Peking University Third Hospital, 49 North Garden Rd., Haidian District, Beijing, 100191 People’s Republic of China; 4 Department of Ophthalmology, Peking University Third Hospital, 49 North Garden Rd., Haidian District, Beijing, 100191 People’s Republic of China; 5 Department of Medical Examination Centre, Peking University Third Hospital, 49 North Garden Rd., Haidian District, Beijing, 100191 People’s Republic of China; 6 Research Center of Clinical Epidemiology, Peking University Third Hospital, 49 North Garden Rd., Haidian District, Beijing, 100191 People’s Republic of China; 7 Department of General Surgery, Peking University Third Hospital, 49 North Garden Rd., Haidian District, Beijing, 100191 People’s Republic of China; 8 Department of Radiation Oncology, Peking University Third Hospital, 49 North Garden Rd., Haidian District, Beijing, 100191 People’s Republic of China

**Keywords:** Nurses, Night shift, Sleep, Metabolism, Risk factors, Cohort studies

## Abstract

**Background and purpose:**

Efforts to improve nurses’ physical and mental health are critical to ensuring the safety and quality of the healthcare system. Long-term studies targeting the relevancy of nurses’ occupation characteristics with health conditions remain insufficient. This study aimed to examine the relationship between nurses’ night shift and sleep problems and metabolic abnormalities risk.

**Methods:**

This study was a part of the National Nurse Health Study, an ambispective cohort study in China, in 2021. Based on an integration physical examination data system, this study carried out a retrospective analysis of 730 nurses from 2018 to 2020 and combined with a questionnaire survey in 2021. The STROBE guidelines were adopted for reporting.

**Results:**

In the 23 (23.0, 24.0) months follow-up, higher night shift load was associated with more sleep problems such as shortened sleep duration, sleep disorders, poor sleep quality, and sleep deprivation. Moreover, night shift load was associated with chronic diseases risk factors, increasing body mass index and body fat, with more night shift density, increasing the occurrence of low levels of high-density lipoprotein cholesterol, high triglyceride, triglyceride/high-density lipoprotein cholesterol ratio, and serum uric acid.

**Conclusion:**

The night shift load has become an occupational health concern, contributing to chronic diseases relevant metabolic risk factors and negative influence on sleep health. Focus on the strategies to improve the sleep quality of nurses undergoing night shift work, optimize work scheduling and ongoing monitor the relevant risk factors are essential to enhance the stability and well-being of the nursing workforce.

***Clinical Trials* registration information:** NCT04572347, on October 1, 2020. https://www.clinicaltrials.gov/ct2/show/NCT04572347

## Background

Nurses are vital in guaranteeing the quality of care in the healthcare delivery system. The efficiency and productivity of nurses play a crucial role in patients' safety and well-being (Kelley et al. 2011). However, increasing numbers of nurses have been suffering from sleep problems in recent years. Many studies have revealed that poor sleep quality is associated with low work performance, productivity loss, efficiency reduction, and even injuries at work (Okajima et al. 2021; Velasco Garrido et al. 2018). Besides, sleep problems are also causes to an array of related physical or mental diseases and illnesses, particularly metabolic-related diseases (Blackwelder et al. 2021; Kecklund and Axelsson 2016). Difficulty falling asleep is associated with cardiovascular disease and a higher risk of all-cause mortality (Robbins et al. 2021). This endogenous shock to the nursing workforce could negatively affect the quality and safety of medical care, patient satisfaction, and overall safety in health facilities (Buerhaus et al. 2007; Xu et al. 2021).

Shift work is defined as work outside of daytime hours, including irregular or rotating schedules and evening and night work (Wang et al. 2011). As a characteristic of the nursing profession, night shift work has many impacts on nurses' performance and health outcomes, particularly on their sleep (Alshahrani et al. 2017; Ganesan et al. 2019; Kim et al. 2013). Findings consistently show that years of shift work negatively impact sleep duration and sleep quality and develop into shift work sleep disorder (Gamble et al. 2011; Ganesan et al. 2019). Although previous research has identified the relationship between shift work and sleep problems, the diversity of relevant long-term research remains insufficient (Kang et al. 2020). Moreover, due to the threat of persistent infectious diseases like COVID-19, influenza, the monkeypox virus pandemic, it is still important to discuss the linkage of the two in the current context of the increased burden on the whole medical system (Al Maqbali et al. 2021).

The more mainstream, long-term cohort studies have orientated the relationship between night shifts and chronic diseases. The UK Biobank cohort study showed that night shift work was associated with higher cardiometabolic multimorbidity (CMM) risk in patients with hypertension, as a coexistence of hypertension and diabetes, coronary heart disease, or stroke. A higher frequency of night shifts (> 10/month) was associated with an increased risk of CMM that was more pronounced for > 10/month in combination with a morning chronotype or lack or prolonged sleep duration (Yang et al. 2022). In the Nurses’ Health Study (NHS), a longer rotating night shift work was associated with the risk of coronary heart disease and type 2 diabetes (Shan et al. 2018; Vetter et al. 2016). Notably, most chronic diseases could be recognized early and prevented by highly relevant metabolic risk factors. Blood pressure, fasting glucose and lipids levels were evaluated as risk factors for cardiovascular disease (Joseph et al. 2017; Lee et al. 2019). Recently, the triglyceride/high-density lipoprotein cholesterol ratio was suggested as predicting the risk of cardiovascular diseases, diabetes and metabolic syndrome (de León et al. 2012; Hadaegh et al. 2009; Shin et al. 2017). More expressively, obesity is the primary risk factor associated with developing a cluster of metabolic disorders, including type 2 diabetes, fatty liver, dyslipidemia, and cardiovascular disease (Yu et al. 2008).

China accounts for about a quarter of the world's nursing workforce and one of the countries facing a shortage of nurses, with relatively long working hours and intensive schedule densities. In the present situation, few cohort studies targeted the relationship between the night shift, sleep problems and metabolic abnormalities of nurses. In order to continuously promote the health of the nursing workforce, a tertiary general hospital-based ambispective cohort study was carried out in 2021. This study aims to examine the relationship between night shift and sleep problems, and metabolic abnormalities of nurses in China, committed to improving nurses’ physical and mental health, optimizing clinical nursing management and maintaining an effective and stable nursing workforce.

## Methods

### Design and participants

**Fig. 1:**
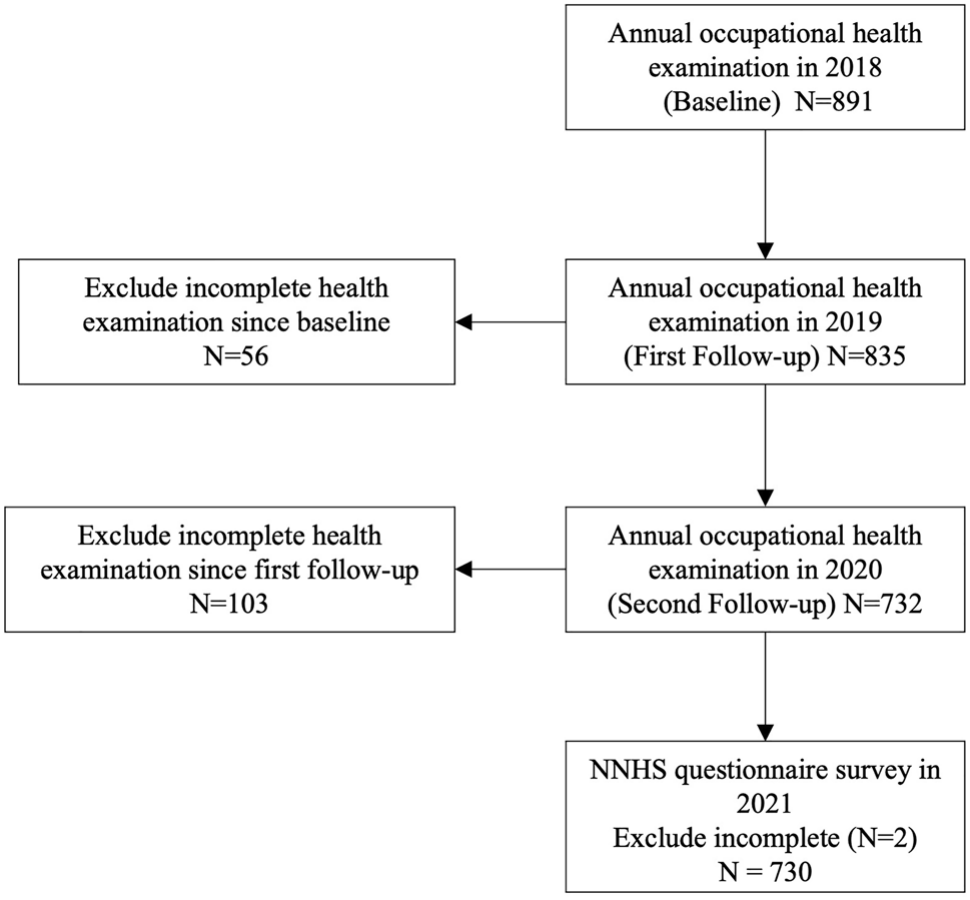
Flowchart Of Study Procedure And Participants Selection From 2018 To 2021. The National Nurse Health Study (NNHS)

This study was a part of an ambispective cohort study in China, registered as the National Nurses Health Cohort Study (NNHS). Following the protocol previously published, the NNHS program was launched on 1 July 2021 (Zhuo et al. 2021). Moreover, utilizing the integrated electronic data system, and considering the potential influence aspect from the time dimension, we carried out a retrospective cohort study from 2018 to 2020, under the framework of NNHS. The start of retrospective time was the first record of occupational health examinations in 2018, and the last follow-up endpoint was defined as the time of the last record in 2020. Besides, a cross-sectional questionnaire survey (the National Nurses Health Cohort Study Self-Report Questionnaire) was accomplished in November 2021 (Fig. 1), to investigate participants’ lifestyle, working status, sleep problems and other psychosocial conditions.

The participants of this study were consistent with NNHS, however, we considered all genders of nurses in our study. We recruited participants from Peking University Third Hospital in Beijing, China, through cluster sampling in 2021. Registered and licensed practical nurses were included, and student nurses, training nurses, and those who failed to undergo annual occupational health examinations, refused to complete the questionnaire and were unwilling to participate were excluded (N = 161) in this study (Fig. 1). The Strengthening the Reporting of Observational Studies in Epidemiology (STROBE) guidelines were adopted for reporting.

## Measures

### Demographic characteristics and lifestyle

Data were collected from the National Nurses Health Cohort Study Self-Report Questionnaire, reported by participants, including age, gender, marital status, nation, religious belief, education, residence, average monthly income, smoking status and alcohol status of lifestyle. The average monthly income was converted into US dollars according to the CNY/US dollar’s mid-point rate in November 2021 (1CNY = 0.15637 US dollars). The survey was conducted through a specific integrated online platform (WJX, Changsha Ranxing Information Technology Co. Ltd) in 2021. All nurse staff had an individual account when login in to the survey platform to avoid repeated participation.

### Night shift work status

This study inquired about the nurses past average night shift work status, in the premise of reviewing the nursing schedule system and ensuring its stability without aberrance over recent years. These data were collected from the National Nurses Health Cohort Study Self-Report Questionnaire in 2021. We queried the extent of exposures to employment years, average night work numbers per month, night shift working hours and patients cared number per night shift. Night shift density was reflected by average night work numbers per month. Night shift density, night working hours and patient care numbers per night shift were scaled into four groups, from less to more. Night shift load was constructed for a comprehensive view of night shift-related factors, and also scaled into four groups by quartiles.

### Sleep problems

Sleep duration, sleep quality, sleep disorder, difficulty falling asleep and sleep deprivation were only reflected in the participants' recent three months sleep situation. Sleep problems were measured by Pittsburgh Sleep Quality Index (PSQI) scale to identify poor sleep quality and sleep disorder (Buysse et al. 1989). This scale was the most commonly used measure of self-report sleep quality and had good reliability and validity among the Chinese population, composed of 7 dimensions that sleep time, sleep duration, sleep efficiency, sleep disturbances, self-rated sleep quality, hypnotic drug use and daytime dysfunction (Zheng et al. 2016). The 19 items (15 items rated 0 to 3) of the PSQI scale were used to evaluate sleep quality as perceived by the participants (Buysse et al. 1989). The participants also self-reported sleep duration, difficulty falling asleep and sleep deprivation. These data were collected from the National Nurses Health Cohort Study Self-Report Questionnaire in 2021.

### Metabolic abnormalities

The health examinations contained disease history taking, blood tests, and physical examinations conducted by physicians. Hyperlipidemia, hypertension, diabetes, thyroid disease and cardiovascular disease and other diseases that relevant to cardiometabolic disease was inquired by physicians and recorded. These data were retrieved from the hospital’s electronic system (Tianrui Kangjian Information Technology Co., Ltd., China). Metabolic factors abnormalities were defined as follows: (1) Body Mass Index (BMI): overweight ≥ 24.0 kg/m^2^, obesity ≥ 28.0 kg/m^2^; (2) High Body Fat: ≥ 25%; (3) High blood pressure: systolic blood pressure ≥ 140 mmHg or diastolic blood pressure ≥ 90 mmHg; (4) High fasting glucose: venous plasma glucose concentration ≥ 6.1 mmol/L; (5) High total cholesterol (TC): fasting venous plasma TC concentration ≥ 5.18 mmol/L; (6) High triglyceride (TG): fasting venous plasma TG concentration ≥ 1.7 mmol/L; (7) High low-density lipoprotein cholesterol (LDL-C): fasting venous plasma LDL-C concentration ≥ 3.64 mmol/L; (8) Low high-density lipoprotein cholesterol (HDL-C): fasting venous plasma HDL-C concentration ≤ 1.04 mmol/L; (9) TG/HDL-C ratio abnormality is defined as above average level of the sample (> 0.866). (10) High uric acid: fasting serum uric acid ≥ 420umol/l for males and ≥ 360umol/l for females. All participants completed the blood sample tests and the missing value of BMI was imputed by average. The health examination data were documented in electronic data files at the end of every year from 2018 to 2020.

### Statistical analysis

Descriptive statistics were calculated to summarize the demographic characteristics of the participants. We further examined the differences in demographic characteristics, working status characteristics, and baseline metabolic factors abnormalities between four scales of night shift density among nurses using the Mann–Whitney U tests and Kruskal–Wallis H tests. The analytic hierarchy process was used to construct the new variable as night shift load. To explore the relationship between night shift load and sleep problems, the binary logistic regression analysis was used to examine average sleep duration, difficulty falling asleep, sleep disorder, sleep quality and sleep deprivation, respectively. The association between night shift density, night shift load and metabolic abnormalities was examined by Cox Proportional Hazards Model. We further included age, gender, marital status, employment years and baseline metabolic abnormalities in the adjusted models. All data were linked and analyzed using IBM SPSS Statistics 24.0 software, with a two-sided significance threshold of p < 0.05.

## Results

In this study, a total of 730 nurses were included in the final sample for analysis (Fig. 1). Subjects were 31.0 (27.0, 36.0) years old, and most were female (95.1%). From the Nurses Health Cohort Study Self-Report Questionnaire results in 2021, 50.8% of the subjects were examined with shortened average sleep duration, 88.2% with sleep disorder, and 79.9% had poor sleep quality. 63.4% of the subjects reported suffering from sleep deprivation, and difficulty falling asleep (28.5%). In the 23 (23.0, 24.0) months follow-up, 9% of the subjects developed high blood pressure, overweight and obesity (11.4%), an increase of body fat (19.6%), fasting glucose (1.5%), TC (19.6%), TG (12.1%), LDL-C (13.9%), serum uric acid (7.0%), and decrease of HDL-C (7.2%).

### Demographic, working status and metabolic characteristics

We found the differences in demographic, working status and metabolic characteristics of the subjects with four scales of night shift density (Table 1). Age, gender, marital status, employment years, smoking status, average night shift working hours and the average number of patients cared for per night shift are statistically significant with the night shift density. As for the metabolic abnormalities at baseline, only total cholesterol appeared to correlate significantly with the night shift density (Table 1).

**Table 1:** Demographic Characteristics And Metabolic Abnormalities Of Study Participants With Different Scales Of Night Shift Density [N (%), median (P_25_, P_50_)]

Characteristics		Night shifts density				χ^2^	*P*
		1–5	6 –10	11 –15	16 –		
Age [median (P25, P50)]		34.0 (29.0, 39.0)	28.0 (25.0, 31.0)	28.0 (25.0, 32.0)	29.0 (28.0, 33.0)	117.335	< 0.01^**^
Gender (N and %)	Men	9 (2.2)	4 (3.2)	22 (12.2)	1 (14.3)	29.344	< 0.01^**^
	Women	408 (97.8)	122 (96.8)	158 (87.8)	6 (85.7)		
Marital status (N and %)	Married	96 (23.0)	67 (53.2)	91 (50.6)	2 (28.6)	63.72	< 0.01^**^
	Unmarried	321 (77.0)	59 (46.8)	89 (49.4)	5 (71.4)		
Employment years [median (P_25_, P_50_)]		16.0 (10.0, 21.0)	9.0 (7.0, 13.0)	9.0 (6.25, 14.0)	11.0 (8.0, 16.0)	113.108	< 0.01^**^
Nation (N and %)	Han nationality	−	111 (88.1)	166 (92.2)	5 (71.4)	1.371	0.712
	Ethnic minority	14 (3.3)	9 (7.2)	10 (5.5)	1 (14.3)		
Religious belief (N and %)	No	408 (370.0)	89 (98.4)	178 (98.9)	6 (85.7)	6.508	0.089
	Yes	9 (14.0)	3 (1.6)	2 (1.1)	1 (14.3)	3.718	
Education (N and %)	Secondary specialized school	3 (0.7)	–	–	–		0.294
	Junior college	92 (22.1)	25 (19.8)	31 (17.2)	3 (42.9)		
	Undergraduate	317 (76.0)	100 (79.4)	149 (82.8)	4 (57.1)		
Residence (N and %)	Living alone	36 (8.6)	30 (23.8)	31 (17.2)	1 (14.3)	0.972	0.81
	With spouse	303 (72.7)	61 (48.4)	101 (56.1)	4 (57.1)		
	Living with parents	60 (14.4)	32 (25.4)	41 (22.8)	2 (28.6)		
	Living with relatives and friends	18 (4.3)	3 (2.4)	7 (3.9)	–		
Average monthly income (N and %)	$625 and below	1 (0.2)	–	–	–	2.075	0.56
	$625 –$1250	41 (9.8)	20 (15.9)	–	1 (14.3)		
	$1250 –$1876	235 (56.4)	66 (52.4)	116 (64.4)	4 (57.1)		
	$1876 and above	140 (33.6)	40 (31.7)	48 (26.7)	2 (28.6)		
Smoking status (N and %)	No	400 (95.9)	117 (92.9)	174 (96.7)	3 (42.9)	49.811	< 0.01^**^
	Yes	5 (1.2)	–	2 (1.1)	1 (14.3)		
	Stop smoking	2 (0.5)	2 (1.6)	1 (0.6)	–		
	Passive smoking	10 (2.4)	7 (5.6)	3 (1.7)	3 (42.9)		
Alcohol status (N and %)	No	375 (89.9)	110 (87.3)	157 (87.2)	5 (71.4)	3.29	0.35
	Yes	40 (9.6)	16 (12.7)	21 (11.7)	2 (28.6)		
	Stop drinking alcohol	2 (0.5)	–	2 (1.1)	–		
Working status							
Night shift working hours (N and %)	≤ 8	314 (75.3)	32 (25.4)	41 (22.8)	–	176.164	< 0.01^**^
	9 –12	72 (17.3)	66 (52.4)	109 (60.6)	4 (57.1)		
	12 –16	29 (7.0)	27 (21.4)	25 (13.9)	2 (28.6)		
	16 –	2 (0.5)	1 (0.8)	5 (2.8)	1 (14.3)		
Number of patients cared per night shift(N and%)	1 –8	251 (60.2)	30 (23.8)	62 (34.4)	2 (28.6)	72.205	< 0.01^**^
	9 –16	42 (10.1)	17 (13.5)	27 (15.0)	1 (14.3)		
	17 –24	46 (11.0)	23 (18.3)	24 (13.3)	4 (57.1)		
	25 –	78 (18.7)	56 (44.4)	67 (37.2)	–		
Metabolic abnormalities							
BMI classification	Light	27 (6.5)	8 (6.3)	13 (7.2)	1 (14.3)	2.486	0.478
	Normal	276 (66.2)	88 (69.8)	112 (62.2)	2 (28.6)		
	Overweight	84 (20.1)	24 (19.0)	43 (23.9)	3 (42.9)		
	Obesity	30 (7.2)	6 (4.8)	12 (6.7)	1 (14.3)		
Body mass	Yes	207 (49.6)	67 (53.2)	101 (56.1)	4 (57.1)	1.11	0.775
High blood pressure	Yes	24 (5.8)	4 (3.2)	11 (6.1)	–	2.353	0.503
High glucose	Yes	12 (2.9)	1 (0.8)	4 (2.2)	–	2.031	0.566
High total cholesterol	Yes	81 (19.4)	18 (14.3)	20 (11.1)	3 (42.9)	10.217	0.017^*^
High triglyceride	Yes	42 (10.1)	12 (9.5)	20 (11.1)	1 (14.3)	0.354	0.95
Low HDL-C	Yes	35 (8.4)	9 (7.1)	15 (8.3)	1 (14.3)	0.554	0.907
High LDL-C	Yes	20 (4.8)	7 (5.6)	18 (10.0)	1 (14.3)	6.636	0.084
High uric acid	Yes	23 (5.5)	6 (4.8)	17 (9.4)	1 (14.3)	4.587	0.205
Self-reported disease history							
Hyperlipidemia	Yes	1 (0.2)	–	–	–	0.751	0.861
Hypertension	Yes	9 (2.2)	1 (0.8)	–	–	4.819	0.186
Diabetes	Yes	5 (1.2)	–	–	–	3.774	0.287
Thyroid disease	Yes	12 (2.9)	–	10 (5.6)	–	1.367	0.242
Cardiovascular disease	Yes	1 (0.2)	–	–	–	0.632	0.427

Body mass index (BMI), high-density lipoprotein cholesterol (HDL-C), low-density lipoprotein cholesterol (LDL-C)

*P < 0.05, **P < 0.01

### Night shift load and sleep problems

**Fig. 2:**
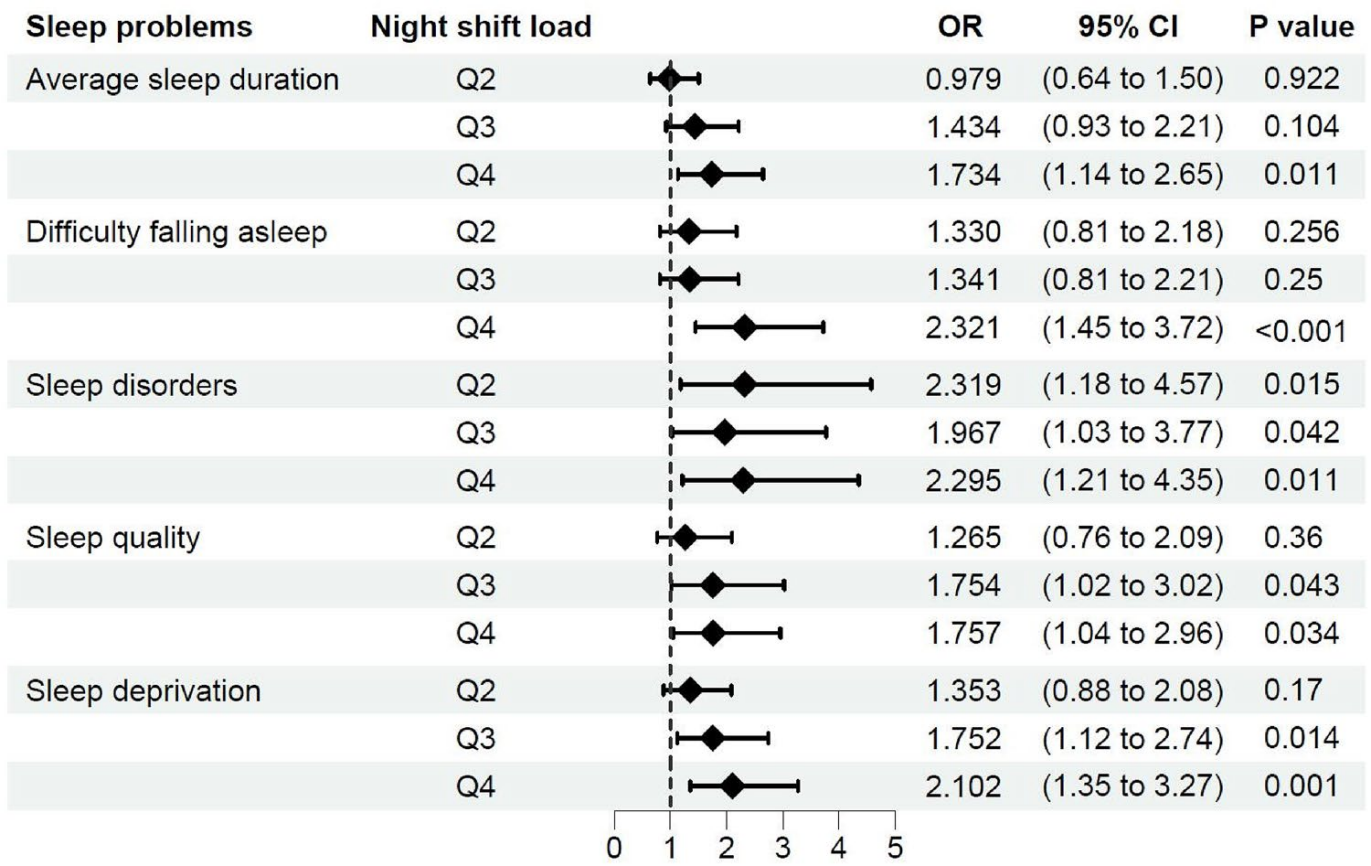
The Odds Ratio And 95% CI Of Age-Gender Adjusted Binary Logistic Regression Models Of Nurses’ Sleep Problems And Night Shift Load**.** Ref: Q1 (< 0.0) of Night Shift Load as reference, Q2 (0.0–0.49), Q3 (0.49–0.83), Q4 (> 0.83). Confidence intervals (CI), odds ratio (OR)

In the age-gender adjusted binary logistic regression models of nurses’ sleep problems, night shift load is the risk factor of shortened average sleep duration (OR = 1.734, 95% CI = 1.137–2.646, p = 0.011), difficulty falling asleep (OR = 2.321, 95% CI = 1.448–3.721, p < 0.001), sleep disorders (OR = 2.319, 95% CI = 1.177–4.568, p = 0.015; OR = 1.967, 95% CI = 1.026–3.771, p = 0.042; OR = 2.295, 95% CI = 1.21–4.35, p = 0.011), poor sleep quality level (OR = 1.754, 95% CI = 1.018–3.024, p = 0.043; OR = 1.757, 95% CI = 1.044–2.958, p = 0.034), and sleep deprivation (OR = 1.752, 95% CI = 1.119–2.743, p = 0.014; OR = 2.102, 95% CI = 1.351–3.269, p = 0.001) (Fig. 2).

In the age-gender adjusted model of average sleep duration, female nurses have a lower risk of less average sleep duration than male nurses (OR = 0.37, 95% CI = 0.174–0.783, p = 0.009). Age contributes to sleep disorders, with a trend of higher risk among elder nurses (OR = 3.308, 95% CI = 1.207–9.067, p = 0.02) (Fig. 2).

### Night shift load and metabolic abnormalities risk

In the age, gender, marital status, employment years and baseline metabolic abnormalities adjusted models (Table 2, Model 3), night shift load has a hazard trend with BMI (HR = 2.68, 95% CI = 1.16- 6.16; HR = 3.07, 95% CI = 1.33–7.12, *P*_*trend*_ = 0.008) and body fat (HR = 2.05, 95% CI = 1.02–4.16, *P*_*trend*_ = 0.037). Night shift density has a hazard trend per five shifts with other metabolism factors (Table 2), including HDL-C (HR = 3.42, 95% CI = 1.19–9.82; HR = 8.71, 95% CI = 1.08–70.43,* P*_*trend*_ = 0.006; for the 11 –15, 16 –group of night shift density), TG/HDL-C ratio (HR = 3.43, 95% CI = 1.06–11.10,* P*_*trend*_ = 0.019) and serum uric acid (HR = 4.64, 95% CI = 1.48–14.55; HR = 4.42, 95% CI = 1.26–15.53; HR = 16.12, 95% CI = 1.29–201.82,* P*_*trend*_ = 0.016; for the 6 –10, 11 –15, 16 –group of night shift density, respectively). Besides, the highest group of night shift density also shows the risk of high blood pressure in model 2 (HR = 6.108, 95% CI = 1.042–35.795, p = 0.045), however, no significant association is observed in the adjusted model followed.

**Table 2:** The Night Shift Density, Night Shift Load And Risk Of Metabolic Abnormalities In Cox Proportional Hazards Models [HR (95% CI)]

Exposure variables		BMI	Body fat	HDL-C	TG/HDL-C ratio	Serum uric acid
Model 1	N	703	724	661	725	669
Night Shift Density^a^	6 –10	0.52 (0.26, 1.05)	**0.48 (0.25, 0.91)**	0.78 (0.27, 2.25)	1.13 (0.70, 1.80)	**2.73 (1.06, 7.02)**
	11 –15	0.63 (0.33, 1.19)	0.87 (0.50, 1.49)	1.77 (0.74, 4.24)	**1.63 (1.04, 2.57)**	**3.16 (1.15, 8.74)**
	16 –	1.16 (0.14, 9.61)	1.34 (0.28, 6.58)	3.98 (0.68, 23.42)	2.78 (0.91, 8.51)	8.98 (0.88, 91.30)
	*P*_trend_	0.203	0.781	0.08	0.016*	0.019*
Night Shift Load (median [range])^b^	Q2	2.01 (0.97, 4.18)	0.95 (0.59, 1.54)	1.30 (0.48, 3.49)	1.35 (0.90, 2.02)	1.71 (0.61, 4.85)
	Q3	**2.81 (1.28, 6.16)**	0.87 (0.48, 1.57)	2.30 (0.84, 6.30)	1.26 (0.79, 2.02)	0.98 (0.29, 3.32)
	Q4	**3.17 (1.46, 6.91)**	1.58 (0.92, 2.74)	1.70 (0.57, 5.04)	0.99 (0.59, 1.65)	1.21 (0.36, 4.10)
	*P*_trend_	0.005*	0.118	0.357	0.824	0.907
Model 2	N	703	724	661	725	669
Night Shift Density^a^	6 –10	**0.46 (0.22, 0.93)**	**0.44 (0.23, 0.85)**	0.74 (0.26, 2.12)	1.12 (0.70, 1.79)	**2.72 (1.05, 7.03)**
	11 –15	0.57 (0.30, 1.08)	0.82 (0.47, 1.41)	1.68 (0.70, 4.05)	**1.62 (1.02, 2.55)**	**3.12 (1.12, 8.70)**
	16 –	1.03 (0.12, 8.65)	1.25 (0.26, 6.09)	3.45 (0.57, 20.73)	2.72 (0.89, 8.36)	8.74 (0.86, 89.39)
	*P*_trend_	0.128	0.671	0.104	0.019*	0.023*
Night Shift Load (median [range])^b^	Q2	**2.09 (1.01, 4.33)**	0.99 (0.61, 1.60)	1.44 (0.53, 3.87)	1.36 (0.91, 2.05)	1.76 (0.62, 4.97)
	Q3	**3.27 (1.48, 7.23)**	0.94 (0.51, 1.70)	2.59 (0.93, 7.16)	1.28 (0.80, 2.06)	1.02 (0.30, 3.48)
	Q4	**3.69 (1.67, 8.17)**	1.75 (1.00, 3.05)	1.88 (0.62, 5.65)	1.01 (0.60, 1.69)	1.24 (0.36, 4.22)
	*P*_trend_	0.002*	0.06	0.296	0.892	0.923
Model 3	N	631	648	591	649	599
Night Shift Density^a^	6 –10	0.52 (0.24, 1.12)	0.51 (0.24, 1.05)	1.19 (0.38, 3.77)	1.15 (0.70, 1.90)	**4.64 (1.48, 14.55)**
	11 –15	0.66 (0.33, 1.31)	0.96 (0.50, 1.84)	**3.42 (1.19, 9.82)**	1.65 (1.00, 2.74)	**4.42 (1.26, 15.53)**
	16 –	1.11 (0.13, 9.69)	0.90 (0.15, 5.57)	**8.71 (1.08, 70.43)**	**3.43 (1.06, 11.10)**	**16.12 (1.29, 201.82)**
	*P*_trend_	0.346	0.155	0.006*	0.019*	0.016*
Night Shift Load (median [range])^b^	Q2	1.56 (0.71, 3.41)	1.10 (0.59, 2.03)	1.90 (0.60, 6.02)	1.29 (0.82, 2.04)	1.10 (0.29, 4.15)
	Q3	**2.68 (1.16, 6.16)**	0.89 (0.41, 1.94)	1.46 (0.40, 5.29)	1.03 (0.62, 1.73)	0.54 (0.12, 2.45)
	Q4	**3.07 (1.33, 7.12)**	**2.05 (1.02, 4.16)**	1.14 (0.29, 4.50)	0.74 (0.41, 1.32)	0.78 (0.18, 3.42)
	*P*_trend_	0.008*	0.037*	0.846	0.206	0.604

Body mass index (BMI), confidence intervals (CI), hazard ratio (HR), high-density lipoprotein cholesterol (HDL-C), triglyceride (TG)

a: 1 –5 of Night Shift Density as reference; b: The test for trend is based on variables containing the median value for each region cut by quartile (median [range]), Q1 (0.0 [< 0.0]) of Night Shift Load as reference, Q2 (0.31 [0.0–0.49]), Q3 (0.67 [0.49–0.83]), Q4 (1.05 [> 0.83])

Model 1: Adjusted for age, gender, smoking status and alcohol status

Model 2: Adjusted for age, gender, smoking status, alcohol status, marital status and working years

Model 3: Adjusted for age, gender, smoking status, alcohol status, marital status working years and baseline metabolic risk factors

*P_trend_ < 0.05, the bold style marked as 95% CI has statistical significance

## Discussion

At present, many countries and regions across the world have successively carried out cohort studies focused on nurses’ health issues. Relevant cohort studies conducted in Asia, such as Japan and South Korea, have mainly addressed the impact of lifestyle, occupational, environmental, reproductive health history, use of female hormone agents, gynecological tumor and other risk factors on women’ s health (Hayashi et al. 2007; Kim et al. 2017). There are also studies on the dynamics of the workforce of nurses and their health (Sawaengdee et al. 2016). Cohort studies in Europe and the United States have investigated the physical and mental health risk of shift work among nurses, yet still, few studies targeted sleep health. A prospective cohort study in Finland showed that continuous shift work with night shifts was associated with increased fatigue during free days (risk ratio = 1.38, 95% CI 1.17 to 1.63) and long sleep (RR = 8.04, 95% CI 2.88 to 22.5) after 6-year follow-up (Härmä et al. 2019). Another study indicated that circadian disruption and older age put rotating shift workers, especially those who work nights, at increased risk of developing clinically significant sleep problems (Tucker et al. 2021).

Relative to developed countries in Europe and the United States, China has a larger number of nursing staff and has become an essential component of the global nursing workforce. As of 2021, there were 5.018 million nurses registered in China, corresponding to a ratio of 3.56 nurses per 1000 people, accounting for nearly 20% of global nurses (News 2022). However, the raised speed of the nursing workforce is far from enough with the growing medical and healthcare demands. Nurses are still under the burden of consecutive and intensive shifts and a high frequency of delays from work (Liang et al. 2017). Up to 69.7% of nurses had at least one type of sleep disorder (Zhang et al. 2016). Moreover, in the context of this study, the work tasks to prevent and control the COVID-19 pandemic brings nurses more stress and sleep problems. Studies conducted in Wuhan showed that 35.06% of nurses were in fatigue status, and the weekly night shift had a low positive correlation with nurses' fatigue (P < 0.01) (Zhan et al. 2020). And 60% of nurses had poor sleep quality, which is significantly associated with depression symptoms (OR = 3.24, 95% CI 1.19 to 8.79) (Tu et al. 2020).

Our findings have shown that nurses with heavier night shift loads (Q3 and Q4 group, with more night shifts per month, workload and working hours) have a higher risk of sleep problems, such as shortened average sleep duration, difficulty falling asleep, sleep disturbances, sleep deprivation and poor sleep quality. This is similar to the results of studies in Italy (Alfonsi et al. 2021; Huang et al. 2021). Night shifts can lead to sleep problems in individuals with disordered circadian rhythms (Cappadona et al. 2021). Sleep disorders will aggravate nurse fatigue and increase the incidence of nursing error accidents (Querstret et al. 2020). The latest studies have shown that sleep disturbance also induces a stress response and disrupts the healthy functioning of the gut microbiota, triggering an inflammatory state (Lopez-Santamarina et al. 2023). This provides support for explaining the effects of night shifts on health at the micro insight. Therefore, nursing managers should evaluate nurses’ night shift load and provide relevant policies and workforce support for departments with heavy night shift loads to improve nurses’ sleep quality.

In the adjusted models of sleep problems with night shift load, our results have also suggested that gender affects the average sleep duration. Male nurses have a higher risk of having shorter average sleep duration than female nurses. This is similar to the results of a cross-sectional study in the United States (Dietch et al. 2017), which showed that the average sleep duration of men was 34 min less than that of women, and this could be affected by distinct reasons, as changes in financial and employment status for males, while emotions, psychological distress for females (Pengo et al. 2018). Future research would take an interest to focus on the sleep quality of nurses of different genders. Our findings that the relationship between age and sleep disorders is consistent with previous research (Morin and Jarrin 2022). With the increase in age, the incidence of sleep disorders is higher due to the effect of physiological, psychological and environmental factors, like less energy and physical strength, anxiety and other negative emotions. Obviously, there is a conflict between their family and backbone roles in the healthcare team. Hence, nursing managers should pay more attention to the elder nurses undergoing shift work and rationalize shift scheduling and human resource adjustments to improve their sleep quality.

Our findings have also contributed to identifying metabolic risk factors associated with night shift load and manifested obesity-related abnormalities. In addition, high total cholesterol in metabolic indicators is statistically significantly related to night shift density. Previous studies have shown that increased high lipoprotein will increase the occurrence of myocardial infarction and stroke events (Holmes et al. 2018), but its relationship with nurses’ night shift load is still unclear. A 20-year prospective cohort study in the United States orientated the relationship between nurse shifts and chronic diseases. Per five-year increment of the duration of rotating night shift work is associated with type 2 diabetes [hazard ratios (HR) = 1.31, 95% CI = 1.19 to 1.44] during 22–24 years of follow-up (Shan et al. 2018). Another study showed a similar association with coronary heart disease risk (HR ≥ 10yrs = 1.34, 95% CI = 1.17 to 1.53) (Vetter et al. 2016). Our finds suggested that night shift load may lead to an increase in BMI and body fat, the latter is a better predictor of cardiovascular risk factors (Zeng et al. 2012). Findings from an 8-year hospital cohort showed that night shift work is associated with a higher risk of developing metabolic syndrome (adjusted OR = 1.36, 95% CI = 1.04 to 1.78) and high waist circumference (adjusted OR = 1.27, 95% CI = 1.07 to 1.78) (Cheng et al. 2021). Other research found more prolonged duration of rotating night shifts was associated with a linear decline in risk of basal cell carcinoma (HR = 0.93, 95% CI = 0.90 to 0.97 per 5-year increase) over ten years of follow-up (Heckman et al. 2017). A Danish Nurse Cohort study found an association between night shift work and mood disorders (HR = 1.31, 95% CI = 1.17 to 1.47) and neurotic disorders (HR = 1.29, 95% CI = 1.17 to 1.42). And these associations were enhanced in nurses with ongoing night shift work (HR = 1.85, 95% CI = 1.43 to 2.39 for mood disorders and HR = 1.62, 95% CI = 1.26 to 2.09 for neurotic disorders) (Jørgensen et al. 2021). The impact of night shifts on illness and health might occur in various ways. Some studies revealed that night shift condition was associated with immune cells expressions and a pro-inflammatory cytokine that display diurnal rhythms and this could be one of the first steps to cardiovascular pathogenesis, represented by biomarkers like IL6, lymphocyte subtypes, neutrophils, NK, and B Cells (Besedovsky et al. 2019; Faraut et al. 2022). The disruption or interruption of the circadian clock-gut microbiota axis due to the night shifts could facilitate gut microbiota disorder that induces inflammatory response (Han et al. 2022; Lopez-Santamarina et al. 2023; Tian et al. 2022). This might lead to the synergistic effect on susceptibility increasing and disease occurrence. Decreasing the night shift load appeals significance of preventing, intervening and treating obesity-related metabolic disease, which potentially developed to chronic diseases.

## Limitation

Some limitations are also noteworthy in this study. First, the night shift status was retrospectively obtained from the questionnaire survey, it might exist recall bias probability. And similar with other self-reported approach, it might exist report bias from the participants, like psychiatric disorders/medication concealment or missed information on purpose for some reason like disease stigma. Besides, the analysis of the relationship between sleep problems and night shift load was based on a cross-sectional survey, inferring causation between the two should further research. In this context, the impacts of the pandemic on sleep problems might have amplified our findings. Second, most of our participants are female staff, and cautions are necessary when applying findings to male staff. Last, our study was carried out in a single center, however, more multicenter research is encouraged to strengthen generalizability within the planned project.

## Conclusion and implications

The risk of sleep problems and cardiometabolic disease in shift workers has been a concern. This cohort study identified the negative effects of night shift frequency and workload on sleep health and metabolic risk among nurses. This is meaningful for the early prevention of some metabolism-related chronic diseases, such as cardiovascular disease, obesity, and diabetes. It is encouraged to focus on the strategies to improve the sleep quality of nurses undergoing night shifts, optimize work scheduling, and ongoing monitoring of metabolic risk factors. Nursing managers should give overall consideration and optimize the night shift duration, frequency, and workload, rather than just focus on the shift schedule. It is encouraged to screen and intervene in the sleep problems and metabolic abnormalities risk of night shift nurses, particularly obesity-related indicators. Supports from policy, administrators and multiple stakeholders at the system level are essential for long-term improvement to enhance the stability and well-being of the nursing workforce.

## Data Availability

The datasets analyzed in this study are available from the corresponding author upon reasonable request.
